# B‐cell depletion limits HTLV‐1‐infected T‐cell expansion and ameliorate HTLV‐1‐associated myelopathy

**DOI:** 10.1002/acn3.52190

**Published:** 2024-08-26

**Authors:** Aowei Lv, Yaofeng Fang, Xiaohong Lin, Jiaying Chen, Huanhuan Song, Ning Wang, Wan‐Jin Chen, Ying Fu, Rui Li, Yi Lin

**Affiliations:** ^1^ Department of Neurology and Institute of Neurology of First Affiliated Hospital, Institute of Neuroscience, and Fujian Key Laboratory of Molecular Neurology Fujian Medical University Fuzhou 350005 China; ^2^ Department of Rehabilitation The First Affiliated Hospital of Fujian Medical University Fuzhou 350005 China; ^3^ Institute of Immunotherapy and Department of Neurology of First Affiliated Hospital Fujian Medical University Fuzhou 350005 Fujian China

## Abstract

**Objective:**

Human T‐cell leukemia virus type 1‐associated myelopathy (HAM) is a chronic, progressive, inflammatory disease with unclear pathogenesis and no effective treatments. We aimed to investigate a novel mechanistic theory and treat HAM patients with rituximab, which can deplete CD20^+^ B lymphocytes in circulation.

**Methods:**

Single‐cell RNA sequencing (scRNA‐seq) data was analyzed to identify HTLV‐1‐associated B cells and their effect on T cells. An observational analysis of our HAM cohort was conducted to elucidate changes in the immunological microenvironment of these patients. Peripheral blood mononuclear cells (PBMC) from HAM patients were isolated to explore the efficacy of B cell depletion in vitro. To assess the effect of B‐cell depletion on HAM patients, eligible participants in our cohort received rituximab therapy (NCT04004819).

**Results:**

ScRNA‐seq results suggest a significant effect of HTLV‐1‐associated B cells on T cells. Additionally, HTLV‐1 was found to infect B cells and depletion of B cells inhibited the proliferation of T cells. Number of B cells in HAM patients had positive correlation with the proviral load and infected cell counts. Depletion of B cells led to a reduction in HTLV‐1 proviral load in vitro. Furthermore, in clinical trial, 14 HAM patients were enrolled. Three patients (21.4%) who received rituximab failed to achieve remission, compared to 24 (85.7%) patients received any other therapy that failed to achieve remission. With a low level of circulating B cells, the proportion of Ki67‐positive cells in CD4^+^ T cells fell.

**Interpretation:**

This study provided evidence that depleting B‐lymphocytes is an innovative strategy for treating patients with HAM and broadens the understanding of the role of B cells in infectious immunity.

## Introduction

The current understanding acknowledges that B cell responses are influenced by the surrounding immune microenvironment, and various factors can trigger different functions of B cells, encompassing both antibody‐dependent and antibody‐independent patterns.^1^ For instance, in multiple sclerosis, B cells are thought to exert antibody‐independent functions primarily in the peripheral blood, particularly the capacity of activated B cells to aberrantly induce pro‐inflammatory T cell responses.^2^ However, in diseases caused by viral infections, the interactions between B cells and T cells, specifically the potential role of B cells in regulating T cells, remain unknown.

Human T‐cell leukemia virus type 1 (HTLV‐1)‐associated myelopathy (HAM) is a rare inflammatory disease characterized pathologically by lymphocyte infiltration and proliferation,^3^ leading to pyramidal tract damage, resulting in unremitting and progressive neurological disorders such as spastic paraparesis, neurogenic bladder, and sensory disturbance of the lower extremities.^4^ Immune dysregulation induced by HTLV‐1 infection is believed to drive chronic inflammation and damage in HAM patients.^5^ A robust immune response against HTLV‐1 is observed in both the peripheral blood and the CNS of HAM patients, as evidenced by the high frequency of HTLV‐1–infected CD4^+^ T cells, cytotoxic T lymphocytes targeting HTLV‐1,^6^, ^7^, ^8^ and the high anti‐HTLV‐1 antibody titer.^9^ Anti‐inflammatory, antiviral therapies, and immunotherapy targeting T cells have been tested but it remains difficult to control the disease progression,^10^, ^11^, ^12^ challenging the central role of T cells in the conventional understanding of HAM pathogenesis, so new mechanism theory and therapy are urgently expanded for HAM.

In this study, scRNA‐seq analysis elucidates the role of B cells following HTLV‐1 infection. HTLV‐1 was found to infect B cells and depletion of B cells inhibited the proliferation of T cells in vitro. Rituximab, a monoclonal antibody targeting CD20 on B‐lymphocytes, was chosen as the treatment to demonstrate the effect of B‐cell depletion therapy on HAM and the underlying mechanism. Furthermore, HAM served as a disease model to explore the influence of B cells on T cells in infectious immunity.

## Subjects/Materials and Methods

### Data processing, scRNA‐seq clustering, and cell type annotation

ScRNA‐seq data of PBMCs from patients infected by HTLV‐1 and healthy controls were obtained from the ENA database (PRJEB47382), raw 10× genomics data underwent processing by CellRanger version 7.1.0 to generate Gene‐Barcode matrices of gene expression. The R package Seurat version 4.3.0.1 was used to analyze the data. Subsequent analysis included all PBMCs from donors consisting of four groups, Healthy Controls (HC, *n* = 3) and HTLV‐1–infected patients with the following clinical diagnoses: people living with HTLV‐1 (PLWH, *n* = 4), smoldering lymphoma subtypes (SML, *n* = 3) and adult T cell lymphoma (ATL, *n* = 7). Sample filtering, integration of cells from all sources, batch correction, dimensionality reduction, and clustering were processed as previously described.^13^ All cells were annotated following the method proposed by Hao et al.^14^ and specific T‐cell subclusters that play a central role in HTLV‐1‐associated diseases were identified based on co‐expression of CADM1 gene and FOXP3 gene.^13^ All clusters identified as B cells were annotated to subclusters. T‐cell clonal expansion characteristics were analyzed by TCR‐seq to define T‐cell subpopulations, with cloneType >30 indicating hyperexpanded subclusters, and these cells were re‐annotated with suffix.

### Identification of HTLV‐1‐associated B cells and cell communication analysis

We assessed the distribution of cells from all four donor groups across each B‐cell subcluster to identify HTLV‐1‐associated B‐cell subclusters. For a systematic analysis of HTLV‐1‐associated B cells and T cells communication molecules, we utilized cell communication analysis using the R package CellChat version 1.6.1.^15^

### Enrichment analysis

Upregulated genes of HTLV‐associated B cells were identified using the R package DESeq2. Subsequently, we conducted Gene Ontology (GO) analyses using the R package clusterProfiler.

### Participants

Our study on patients with HAM is divided into two parts: an observational analysis comparing the immunological microenvironment between our patient cohort and healthy controls, and a 48‐week single‐arm rituximab intervention trial involving eligible patients from the cohort. The patient numbers vary between the two study segments.

Patients aged ≥18 years old and diagnosed with HAM based on the World Health Organization guidelines were recruited to our cohort. Screening for HTLV‐1/2 infection was conducted using an ELISA HTLV1/2 kit (Wantai, China), with confirmation of HTLV‐1 infection by testing serum and cerebrospinal fluid (CSF) using a western blot kit (HTLV BLOT 2.4, MP Biomedicals, Germany). Additionally, HTLV‐1 tax and env gene were amplified by nested PCR to confirm the presence of the HTLV proviral genome in these patients' lymphocytes. Patients with an Expanded Disability Status Scale (EDSS) score of 7.0 or less, who were not undergoing current treatment, were eligible for inclusion. Exclusion criteria comprised individuals with serious complications, other chronic infections, a history of allergic reactions to antibody drug products, and severely impaired immune responses. Sixteen age‐ and gender‐matched healthy controls (HC) were recruited from the hospital, all of whom reported no history of neurological diseases and tested seronegative for HTLV antibodies in our laboratory.

### Observational analysis of the immunological microenvironment of HAM patients

For the observational analysis of the immunological microenvironment, we selected an appropriate number of patient samples based on the specific experiments conducted. Initially, PBMCs from patients with HAM were isolated by centrifugation using lymphocyte separation media, numbers of immune cells were assessed using flow cytometry, and HTLV‐1 proviral load in vivo was monitored by droplet digital polymerase chain reaction (ddPCR) assay, neopterin concentrations were measured using an ELISA kit (IBL International, Germany). Then, CD19^+^ B cells were isolated from PBMCs using magnetic beads, and the existence of HTLV‐1 Tax gene in B cells was identified using nested PCR. Additionally, we analyzed the correlation between the number of B cells and the HTLV‐1 proviral load as well as the number of infected T cells using linear regression. As there are no universal markers for HTLV‐1‐infected B cells, we could not assess the frequency of HTLV‐1‐infected B cells.

### Investigation the efficacy of B cell depletion therapy on HAM in vitro

To investigate the mechanism of B cell depletion therapy under a single alternating quantity condition, we established a cell model of rituximab‐induced B cell depletion. To deplete B cells, PBMCs from patients with HAM were cultured in 24‐well round bottom microplates at 2 × 10^6^ cells/well with Roswell Park Memorial Institute (RPMI) 1640 medium (Sigma‐Aldrich, Germany) at temperature 37°C/5% CO_2_. In this study, 100 μg/mL rituximab (Roche, Switzerland) and 200 μL complement were cocultured with PBMCs for 24 h, and PBMCs in the control group were cocultured with PBS. B cell depletion ratio was acquired using a flow cytometer (Beckman Cytoflex S). While carboxyfluorescein diacetate succinimidyl ester (CFSE) (CellTrace CFSE cell proliferation kit; Invitrogen) was performed to investigate the impact of B cell depletion on T cell proliferation according to the manufacturer's instruction. Additionally, HTLV‐1 PVL of cultured cells was assessed using ddPCR.

### Clinical trial study design

The clinical trial was designed as an open‐label, single‐center pilot study to evaluate the efficacy of rituximab for patients with HAM (clinical trial registration identifier NCT04004819). It was conducted at the First Affiliated Hospital of Fujian Medical University in Fuzhou, China, independently initiated and funded by the investigators. Data analysis was conducted by a team of independent investigators at the Fujian Medical University School of Public Health. Rituximab was purchased without discount from Roche, which had no role in any other aspect of the study. The study protocol and informed consent procedures were approved by the Institute Ethics Committee. Written informed consent was provided by the patients and their health care proxies.

### Intervention and follow‐up

Eligible participants received rituximab therapy twice, first during the baseline hospitalization and second during the 24‐week follow‐up period, at a dose of 375 mg/m^2^ of body surface area. Participants will be followed every 12 weeks for 48 weeks. Additionally, following the initial 8 weeks of treatment, we extended an invitation for an additional follow‐up, which only 8 patients attended as scheduled, while the remaining six patients were unable to attend the hospital for the follow‐up.

### Droplet digital PCR (ddPCR)

The QX200 system (Bio‐Rad, USA) was utilized for HTLV proviral load (PVL) quantification using ddPCR. Primer and probe sequences are detailed in Table S1. Negative controls included no template controls and DNA from HTLV‐seronegative biological samples. QuantaSoft software version 1.3.2.0 (Bio‐Rad, CA, USA) was used to quantify the copies/μL of each queried target per well. All samples were run in duplicate, and the PVL is the average of the two measurements. The PVL was calculated as the percentage of infected cells using the following formula.^16^

$$\mathrm{PVL}=\left(\frac{\text{Quantity of HTVL}-1\mathrm{pX}}{\left(\frac{\text{Quantity of}\;\beta -\text{globin gene}}{2}\right)}\right)\times 100$$

### Evaluation and outcomes

Pharmacodynamics were assessed by measuring changes in circulating B cells. The frequency of circulating B cells was monitored using flow cytometry before and after rituximab infusions at each interval. In most cases, the pharmacological effect of rituximab was expected to last for at least 24 weeks; reinfusion was continued if B cells exceeded 1% of total lymphocytes.^17^

The primary outcome was defined as a decrease of 1 point in the pyramidal or bowel‐bladder function score in EDSS. Improvement in either pyramidal function or bowel‐bladder function after 48 weeks of therapy would demonstrate the efficacy of rituximab in HAM patients. Ki67 expression was used to evaluate the proliferation of T cells. The Overactive Bladder Symptom Score (OABSS) was utilized to assess neurogenic bladder symptoms in patients. Changes in muscle tone in both lower limbs of the patients were assessed using the Modified Ashworth Scale (MAS), while improvements in ankle clonus were measured via the Composite Spasticity Index (CSI).

We documented the frequency and severity of adverse events reported by patients. All ongoing adverse events were followed up until resolution or until they no longer required follow‐up according to the protocol. Serious adverse events were monitored by investigators. Adverse events were evaluated according to the National Cancer Institute Common Terminology Criteria for Adverse Events, version 4.0.

### Statistical analysis

Meta‐analysis of previous studies utilizing the EDSS assessment in HAM suggests a 21% rate of improvement among patients following any treatment.^18^, ^19^, ^20^ Therefore, the null hypothesis of this trial is that the overall response proportion is more than or equal to 40%. Sample size was calculated based on the overall response rate with level of significance (*α* = 0.05), 90% power (1−*β* = 0.9), and a dropout rate of 20%, which required a sample size of approximately eight participants. To provide more evidence, we included all participants who completed treatment and their 48‐week follow‐up.

All the data collected in the trial was analyzed by a statistician using SPSS 26.0 software (IBM Corp., Armonk, NY, USA). For baseline or every follow‐up data, discrete variables (EDSS) and continuous variables (PVL, flow cytometry results) were presented as median and interquartile range. *F*‐test was used to assess the homogeneity of variables, while Kolmogorov–Smirnov test was used to evaluate the normality before and after treatment. For normally distributed variables, group differences were assessed by the independent 2‐tailed *t*‐tests. For variables that were not normally distributed, the Wilcoxon Mann–Whitney test was used, as appropriate, or the data were log‐transformed for parametric testing. Benjamini & Hochberg correction was applied to adjust for multiple comparisons.

## Results

### HTLV‐1‐associated B cells communicate with T cells

**Figure 1:**
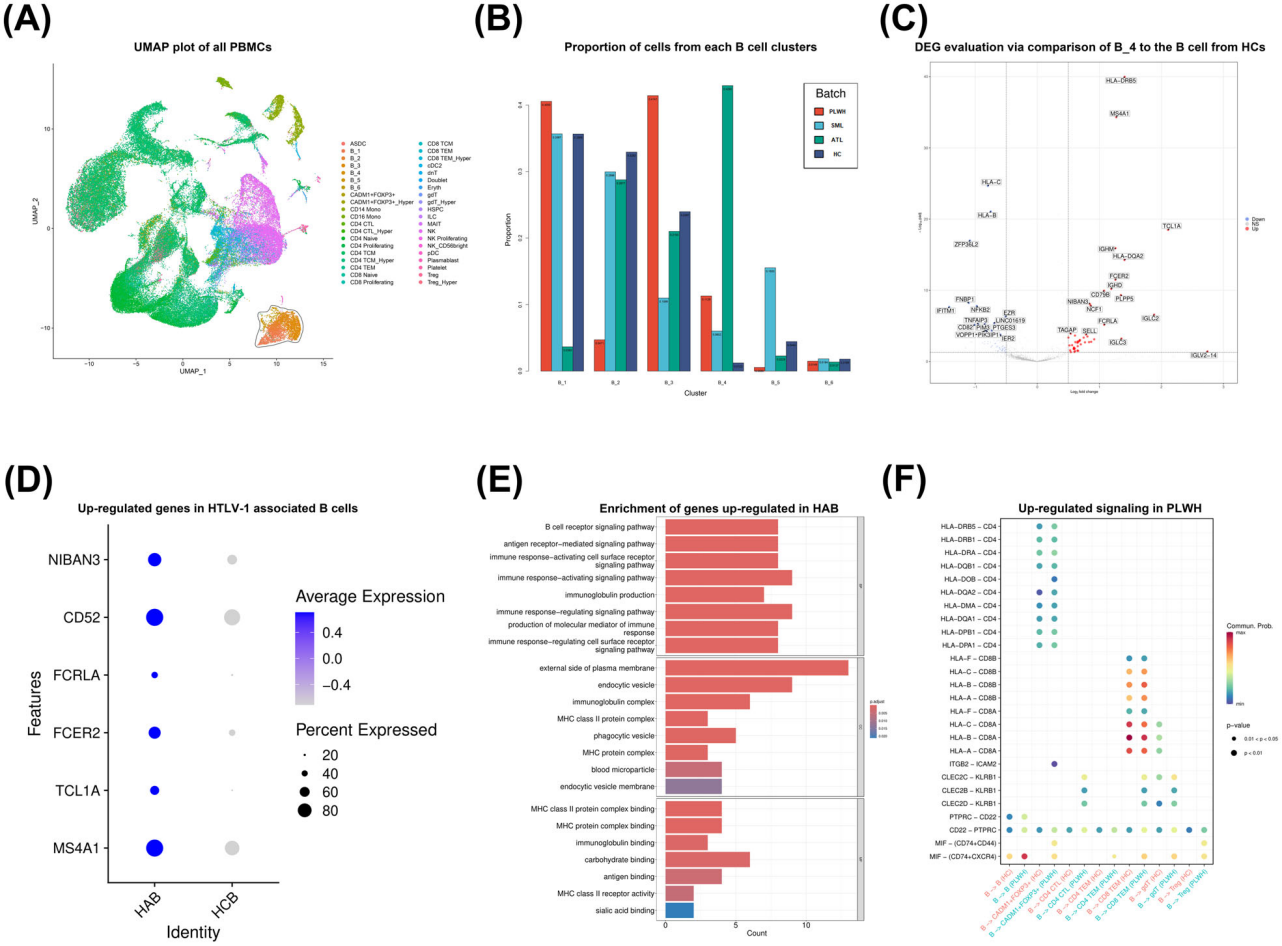
Identification of HTLV‐1‐associated B cells and cell communication of B cells and T cells by scRNA‐seq. (A) Uniform manifold approximation and projection (UMAP) visualization of the integrated scRNA‐seq from all donors, colored by the identified cell cluster. The closed curve formed by black line encircle the subpopulation of B cells. (B) Bar plots representing the proportion of PLWH, SML, ATL, and HC cells out of cells in each of the clusters identified as B cells. (C) Volcano plot representing differentially expressed genes between HTLV‐1‐associated B cells from PLWH and B cells from HCs. (D) Dot plot showing representative up‐regulated genes in HTLV‐1‐associated B cells (HAB) from PLWH compared to healthy control B cells (HCB). (E) Pathway visualization bar plot of up‐regulated genes using the GO biological process database. (F) Bubble plot showing the comparison of the significant ligand‐receptor pairs between PBMCs from HC and PLWH population, which contribute to the signaling from HTLV‐1‐associated B cells to T cells. The colors in the bubble plot are proportional to the communication probability, where blue and red correspond to the smallest and largest values, respectively. Blank space represents no significance. PBMCs from HC population and PLWH population were shown respectively.

Figure 1A illustrates the identification and annotation of a total of 40 cell groups from all donors, mainly focused on T cells and B cells. Given the current absence of reliable marker genes for HTLV‐1‐associated B cell subtypes, B cells were categorized into six subgroups in order to identify the subset most strongly associated with HTLV‐1, as illustrated in Figure S1B. Strikingly, group B_4 represent distinctive B cells observed in patients infected by HTLV‐1 but not in the healthy controls, we define this group as HTLV‐1‐associated B cells (Fig. 1B).

Up‐regulated genes in HTLV‐1‐associated B cells from PLWH were identified using DESeq2 (Fig. 1C, D). Subsequent GO analyses confirmed the upregulation of genes associated with antigen presentation in HTLV‐1‐associated B cells (Fig. 1E). Additionally, we conducted ligand‐receptor analysis using CellChat. Bubble plot shows the comparison of the significant ligand‐receptor pairs between PBMCs from healthy controls and PLWH population, contributing to the signaling from HTLV‐1‐associated B cells to T cells (Fig. 1F). These findings suggest a significant effect of HTLV‐1‐associated B cells on T cells, particularly the CADM1^+^FOXP3^+^ T cells, which were considered to be central in HTLV‐1‐associated diseases.^13^ Specifically, cell communication results predicted that HTLV‐1‐associated B cells could regulate T cells, mainly through antigen presentation by HLA‐II/CD4 and cytokine secretion such as MIF/(CD74^+^CXCR4) and MIF/(CD74^+^CD44).

### HTLV‐1‐associated T cells and CSF inflammatory markers is detectable in HAM but has no impact on B cell counts

**Figure 2:**
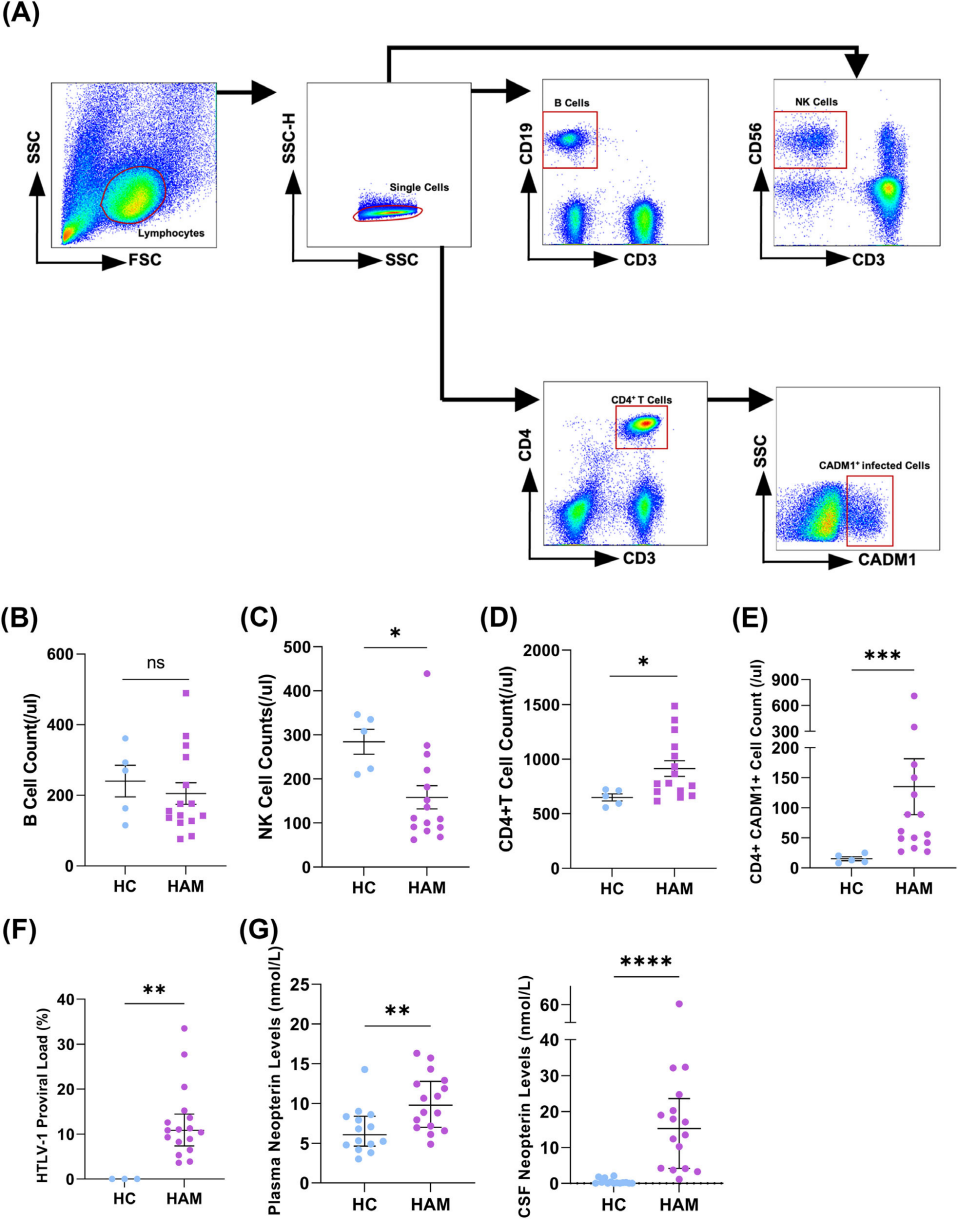
Changes of immune environment and viral burden in HAM patients at baseline. (A) Representative flow cytometry pattern showing the gating strategy of immune cells. (B) Changes of number of B cells n HAM patients (*n* = 15) compared with healthy controls (*n* = 5). (C) Changes in number of NK cells in HAM patients (*n* = 15) compared with healthy controls (*n* = 5). (D) Changes of number of CD4^+^ T cells in HAM patients (*n* = 15) compared with healthy controls (*n* = 5). (E) The number of infected T cells in HAM patients (*n* = 15) compared with healthy controls (*n* = 5) at baseline. (F) HTLV‐1 proviral load in HAM patients (*n* = 15) compared with healthy controls (*n* = 3) at baseline. (G) Changes of neopterin level in plasma (left) and cerebrospinal fluid (right) in HAM patients (*n* = 16) compared with healthy controls (*n* = 14). *P* value was determined by the Mann–Whitney test. ns *P* > 0.05, *P < 0.05, **P < 0.01, ***P < 0.001, ****P < 0.0001.

From January 2015 to January 2019, 25 patients were diagnosed with HAM and enrolled in our cohort. Biological samples from this cohort were utilized for a observational study comparing the immunological microenvironment with healthy controls. Number of immune cells were assessed (Fig. 2A). Compared with healthy controls, the number of B cells showed no changes (156 vs. 270 counts/μL, *P* = 0.553) (Fig. 2B), the number of NK cells decreased (111 vs. 308 counts/μL, *P* = 0.015) (Fig. 2C), while CD4^+^ T cells increased (780 vs. 661 counts/μL, *P* = 0.025) (Fig. 2D). The median of absolute number about CD4^+^CADM1^+^ cell was 61 counts/μL (42–150 counts/μL) (Fig. 2E). The median of HTLV‐1 PVL of PBMCs was 10.84% (7.37–14.44%) (Fig. 2F). Additionally, the Immune environment of HAM patients exhibited significant changes. In addition, neopterin level was significantly higher in the plasma (9.78 (6.70–12.79) vs. 6.06 (4.63–8.41) nmol/L, *P* = 0.007, and CSF (15.29 (4.13–23.62) vs. 0.15(0.00–0.68) nmol/L, *P* < 0.0001)) of HAM patients compared to healthy controls (Fig. 2G).

### HTLV‐1 virus infects B cells and could affect T cells

In classical theory, T cells are the major target of HTLV‐1. We wonder what mechanisms underlie the development of HTLV‐1‐associated B cells following viral infection. And the role HTLV‐1‐associated B cells play in HAM pathogenesis remains unclear.

**Figure 3:**
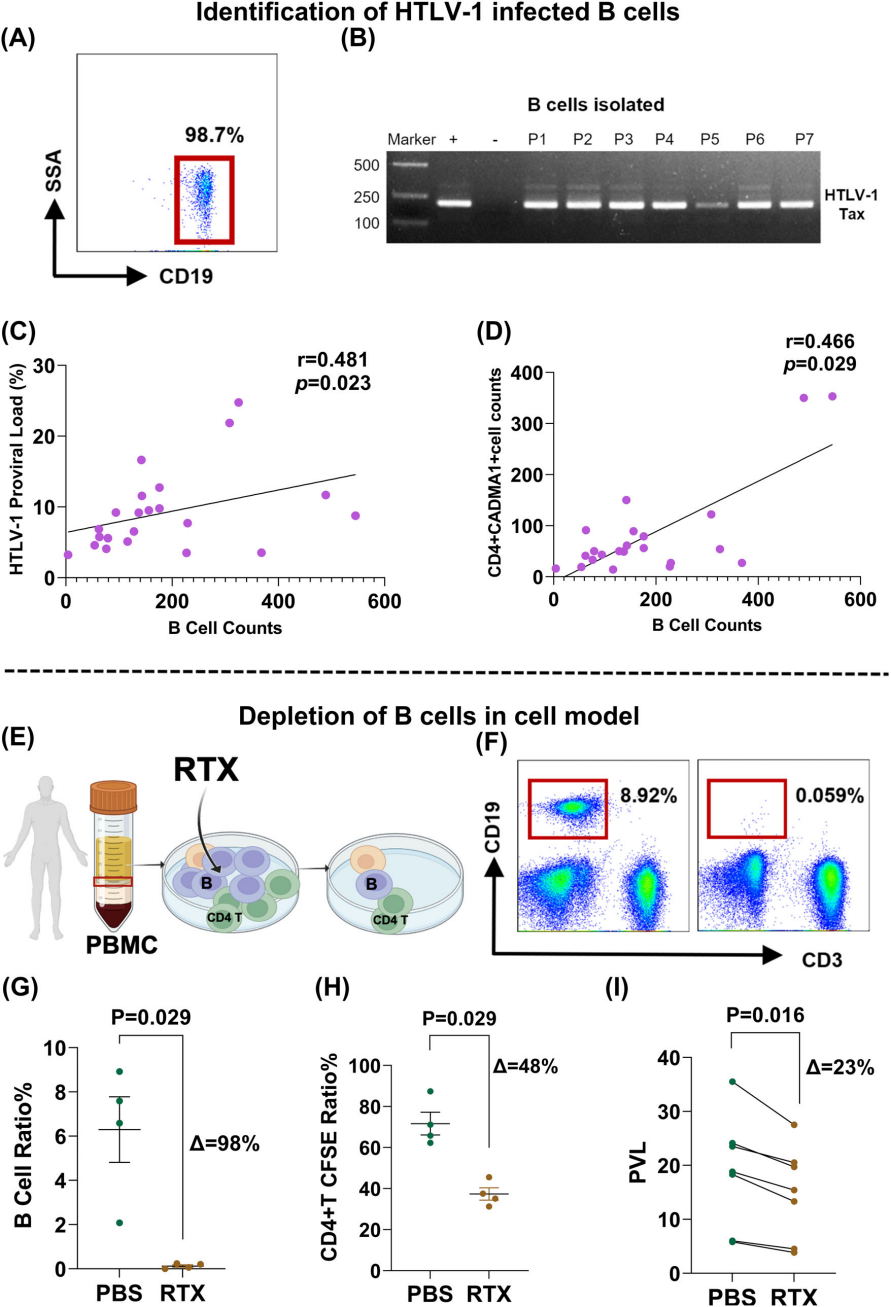
Effect of depleting B cells on virus burden. (A) Peripheral CD19^+^ B cells were isolated from HAM patients by magnetic beads (purity confirmation by flow cytometry routinely >98%). (B) The presence of the HTLV‐1 genome tax in B cells was amplified by nested PCR. (C) Correlation analysis of B cell counts with HTLV‐1 proviral load (*n* = 22). (D) Correlation analysis of B cell counts with the number of infected T cells (*n* = 22). (E) Pattern of B cell depletion cell model in vitro. Rituximab or PBS was cocultured with PBMC isolated from HAM patients to deplete B cells in vitro, aiming to explore the change of proviral load when the existence of B cells is the only variate, and CFSE was used to detect the changes of CD4^+^ T cell proliferation after B cell depletion. (F) Representative flow cytometry plot of B‐cell depletion effect in the cell model. (G) B cell ratio decreased after B‐cell depletion (*n* = 4). *P* value was determined by the Mann–Whitney test. (H) Statistical diagram of CFSE staining of CD4^+^ T cell in the B cell depletion cell model (*n* = 4). *P* value was determined by the Mann–Whitney test. (I) Quantification of the HTLV‐1 proviral load with or without rituximab by digital PCR (*n* = 7). *P* value was determined by Wilcoxon matched‐pairs signed rank test.

B cells from HAM patients were isolated using magnetic beads, achieving a sorting ratio of over 98% as confirmed by flow cytometry (Fig. 3A). HTLV‐1 proviral genome could be amplified in B cells using nested PCR (Fig. 3B), suggesting that B cells could also be infected. Hence, we hypothesized that B cells may act as a reservoir for HTLV‐1. Additionally, linear analysis indicated a positive correlation between the B cells counts and HTLV‐1 proviral load (*r* = 0.481, *P* = 0.023) (Fig. 3C), as well as infected T cell counts (*r* = 0.466, *P* = 0.029) (Fig. 3D).

### B cell determined outcome of HTLV‐1 infection T in vitro test

To study the underlying mechanism of B‐cell depletion therapy under a single influencing factor, a cell model was created in which B cells from HAM patients could be depleted by rituximab after coculture for 24 h (Fig. 3E, F). The results demonstrate effective depletion of B cells (7.09% (4.34–8.26%) vs. 0.13% (0.03–0.22%)) (*P* = 0.05) in this model (Fig. 3G).

Abnormal proliferation of T cells is an important link in the pathogenesis after HTLV‐1 infection.^21^ Proliferation of T cells decreased from 71.7% (64.05–79.25%) to 36.30% (33.20–41.50%) (*P* = 0.029) following B cell depletion in vitro, as evidenced by monitoring CFSE using flow cytometry (Fig. 3H), as well as the HTLV‐1 PVL decreased from 18.80% (12.15–23.80%) to 15.40% (8.90–20.10%) (*P* = 0.016) (Fig. 3I).

### B‐cell‐depletion therapy ameliorate clinical disability of patients with HAM

Our cohort comprises 25 HAM patients, 17 of them underwent screening, while two withdrew from our cohort. The remaining 15 patients were enrolled and scheduled to receive rituximab, yet one did not, resulting in a final analysis of 14 patients. The details are presented in Figure S2A. All 17 patients were proved to be infected by HTLV‐1 using Western Blot and nested PCR (Figure S2B, C). All 17 cases of HAM were from Fujian Province, China, with eight originating from Ningde City. The median age of onset was 44.0 years old (39.5–49.0). Spasticity presented as a common clinical manifestation in all patients. Fourteen out of 17 patients (82%) reported urinary bladder disturbances, characterized by irritative bladder symptoms and urinary incontinence. Nocturia and urinary loss were the most serious concerns. Additionally, eight out of 17 patients (47%) experienced reduced surface sensation. The median EDSS score was 4.0 (3.5–5.5).

**Figure 4:**
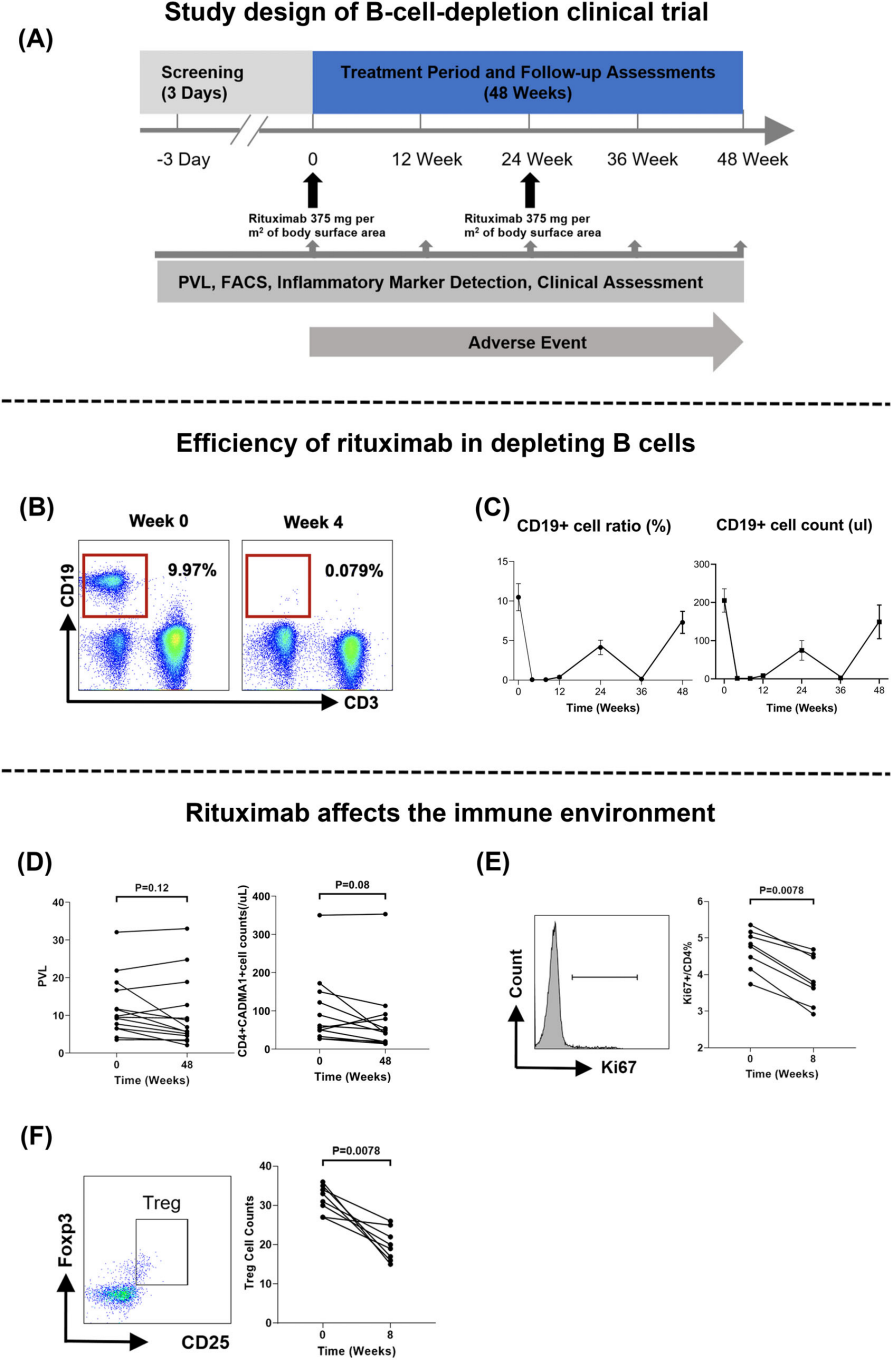
Intervention plan and pharmacodynamics of rituximab. (A) Design of the study. (B) Representative flow cytometry pattern of circulating B cells in peripheral blood. (C) Graph of changing trend in the proportion of B cells in lymphocytes after treatment with rituximab. (left) Changes in the absolute number of B cells after treatment with rituximab. There was a significant depletion of circulating B cells after the first dose of rituximab and rebound at 24 weeks as expected. (right) (D) Individual levels of HTLV‐1 proviral load (left) and the number of infected cells (right) analyzed before and after rituximab treatment. *P* value was determined by Wilcoxon matched‐pairs signed rank test. (E) Representative histogram (left) and statistical diagram (right) of Ki67 expression in CD4^+^ T cells indicating cell proliferation condition. *P* value was determined by Wilcoxon matched‐pairs signed rank test. (F) Representative dot plots (left) and statistical diagram (right) of number of Treg cell. *P* value was determined by Wilcoxon matched‐pairs signed rank test.

Fourteen patients received rituximab therapy twice and underwent the 48‐week follow‐up (Fig. 4A). Adverse events are listed in Table S2. The pharmacodynamic effect of rituximab on CD19^+^ B cell count and ratio was observed within 4 weeks, resulting in a significant and robust depletion of circulating B cells (Fig. 4B). A rebound in CD19^+^ B cell counts was observed at the 24‐week follow‐up after the initial dose of rituximab, as expected. Consequently, rituximab was re‐administered at 24‐week follow‐up, leading to a robust depletion and subsequent rebound at 48‐week follow‐up (Fig. 4C). The observation period for pharmacodynamic effect lasted for 48 weeks.

Depleting B cells in HAM patients tend to decrease HTLV‐1 proviral load and infected T cell counts (HTLV‐1 PVL: 9.64% vs. 6.31%, *P* = 0.12; infected T cell counts/μL: 59 vs. 47, *P* = 0.08) (Fig. 4D). However, depleting B cells in HAM patients inhibited T cell proliferation. Eight weeks after initial administration of rituximab, with a low level of circulating B cells, the proportion of Ki67 positive cells in CD4^+^T cell fell to 3.76% (3.23–4.54%) from 4.81% (4.23–5.14%) (*P* < 0.01) (Fig. 4E). HTLV‐1 infection induces T cells to acquire the phenotype of Treg cell with the expression of FOXP3. Lowering the Treg cell counts may also inhibit the dissemination of HTLV‐1 infection. The count of Treg cells decreased to 20 (16–24) counts/μL from 32 (28–35) counts/μL (*P* < 0.01) when comparing levels 8 weeks after the initial rituximab dose with baseline in this study (Fig. 4F).

**Figure 5:**
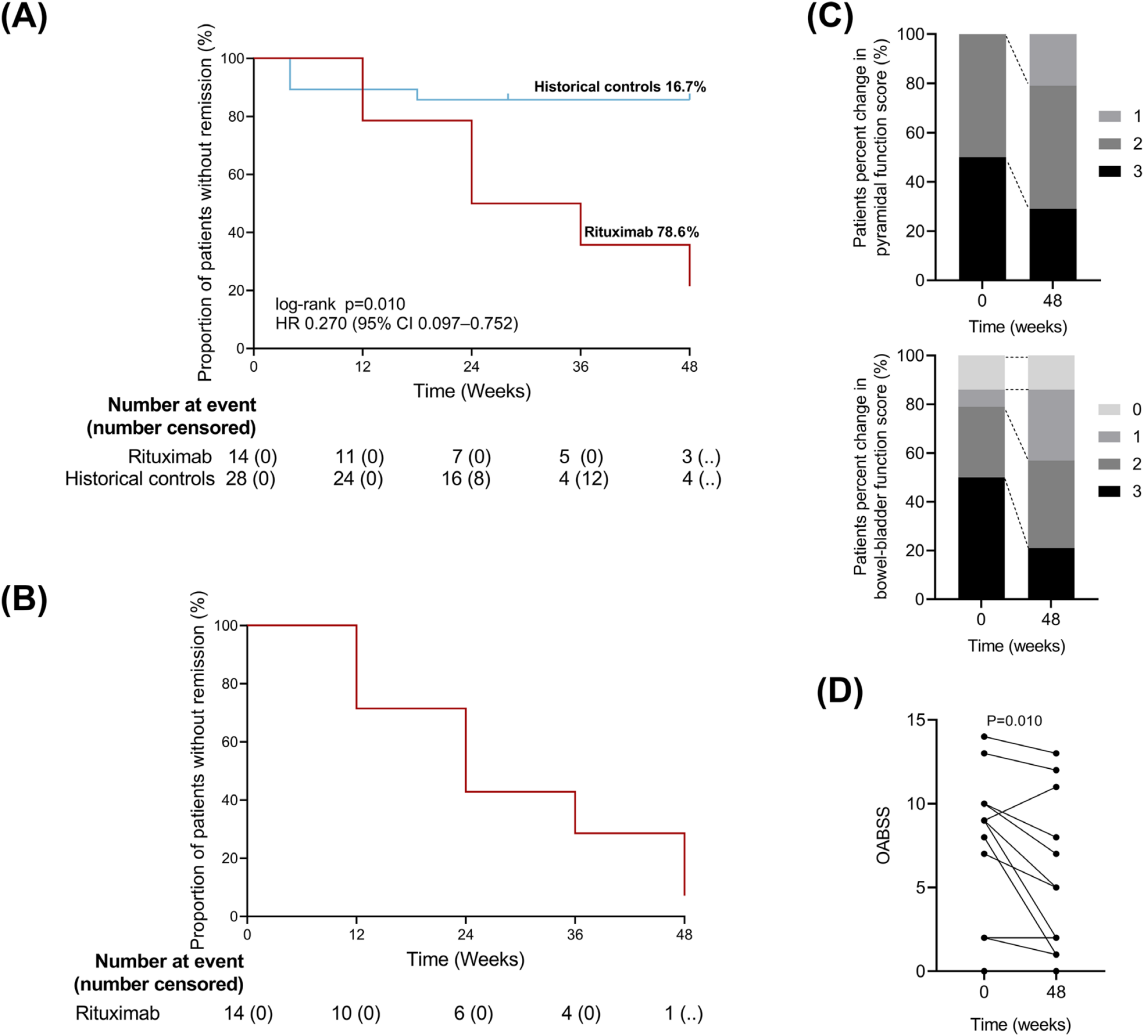
Clinical efficacy of rituximab therapy on HAM patients. (A) Survival analysis for rituximab and historical control treatment to assess EDSS improvement. HR = hazard ratio. (B) The proportion of patients without remission after treatment with rituximab by survival analysis. Remission is defined as a 1‐point improvement in pyramidal function or bowel‐bladder function score. (C) The distributions of 14 patients according to pyramidal function and bowel‐bladder function score at before and after 48 weeks treatment. A value of 100% represents 14 participants. Week 0 represents the day before treatment. Black dotted line indicates the trend of change in each part. (D) Change in OABSS at baseline and 48‐week.

We also investigated the effect of rituximab on clinical disability. During the 48‐week trial period, 3 out of 14 patients (21.4%) who received rituximab failed to exhibit a reduction in the EDSS score, while 24 out of 28 patients (85.7%) who received any other therapies failed to exhibit a reduction in the EDSS score in previous studies (group difference 64.3%, HR = 0.267, 95% CI: 9.6–74.3, log‐rank *P* = 0.008) (Fig. 5A). If either pyramidal function or bowel‐bladder function score decreased one point, which considered to occur event, 13 out of 14 participants (92.8%) receiving rituximab achieved remission during the 48‐week study period (Fig. 5B). Compared to baseline, five patients (35.7%) showed improvement in pyramidal function scores, while eight patients (57.1%) showed improvement in bowel‐bladder function scores. Notably, bowel‐bladder function showed a more pronounced improvement than pyramidal function, with the proportion of patients whose bowel‐bladder function score reached 3 decreased from 50% to 21% (Fig. 5C). Significant improvement in OABSS (*P* = 0.010) indicated relief of neurogenic bladder symptoms in these patients, such as decreased daytime frequency, nocturia, urgency, and urinary incontinence (Fig. 5D). Although our measurements do not confirm changes in lower limb muscle tone in HAM patients (Figure S3C), the assessment of ankle clonus demonstrates significant improvement (*P* = 0.012, Figure S3D).

## Discussion

HAM is a chronic neuroinflammatory disease with significant geographic spread. Reports suggest that Fujian province has been identified as an endemic region for HTLV‐1 and exhibits the highest proportion of HTLV‐1 infection among blood donors in Chinese mainland.^22^ This geographical distribution characteristic forms the practical basis for our research.

HAM, being an aggressive and irreversible degenerative disease, poses challenges in slowing down its progression. Currently, no effective or satisfactory treatments for HAM exist. Despite attempts with glucocorticoids and interferon‐α, targeting CCR4^+^ T cells therapy or targeting CD25^+^ T cells therapy, and interleukin‐15 to suppress inflammation, disease progression remains challenging to manage.^12^, ^19^, ^23^, ^24^ Although a decrease in HTLV‐1 proviral load in cell models was reported, the clinical effectiveness of teriflunomide in HAM patients remains unproved.^21^ While some studies have suggested that B cells may play a pathogenic role in HAM.^25^, ^26^

To enhance comprehension of the pathogenic mechanisms following HTLV‐1 infection and explore novel therapeutic targets, scRNA‐seq data from PBMCs of HTLV‐1 infected patients were obtained and analyzed. HTLV‐1‐associated B cells were identified and considered to play a pathogenic role by regulating T cells after HTLV‐1 infection. Our observational immunological microenvironment analysis also identified immunological abnormalities in our HAM patient cohort. Additionally, we elucidated some of the mechanisms through which B‐cell depletion therapy operates, specifically by directly eliminating infected B cells and by inhibiting T‐cell proliferation. Furthermore, we conducted an open‐label, single‐arm clinical trial that proved the efficacy of B‐cell depletion therapy in patients with HAM, and we provided a summary of immunological characteristics of HAM patients. Distinct from anti‐inflammatory, antiviral therapies, and immunotherapy targeting T cells, this study proposes a new immunotherapeutic strategy targeting B cells.

The current study has several limitations. Primarily, due to the unavailability of scRNA‐seq data from CSF cells in HAM patients, we were unable to analyze changes in immune cells in the central nervous system following HTLV‐1 infection, and the validation of up‐regulated genes in HTLV‐1‐associated B cells, such as TCL1A, was not conducted in this study. Additionally, the small sample size of the clinical trial, combined with its single‐arm nature, resulted in a lack of effective randomization and rigorous control. The observation and follow‐up period were insufficient for chronic progressive diseases, thereby incompletely validating long‐term treatment efficacy. Moreover, inflammatory factors, especially those in CSF, are important biomarkers in the field of neuroinflammation. However, we did not collect the CSF of all 14 patients due to ethical considerations at the final follow‐up time of this study. This prevented us from comparing the changes in cerebrospinal fluid inflammatory factor levels at 48 weeks with the baseline. Lastly, participants were all from Fujian province, China, potentially limiting the generalizability of the results.

Despite these limitations, this study remains meaningful. B cells, traditionally known for their protective role in viral infections through antibody secretion,^27^, ^28^ were identified as contributors to the pathogenesis of HTLV‐1‐associated diseases by promoting abnormal T cell proliferation. This study established a basis for further investigations into B cell responses and the functions of B cell/T cell interactions in infectious immunity. Furthermore, in vitro experiments demonstrated that depletion of B cells mitigated the effects induced by HTLV‐1 infection. This finding was corroborated by the efficacy of rituximab treatment in patients with HAM. After all, this study offers innovative strategies for treating patients with HAM.

## Author Contributions

The study was created by YF. YL and NW secured funding for the study. The clinical trial was designed by YF and YL. The patient was diagnosed and followed up by WJC, YL, and NW. The data was collected and analyzed by AWL, YFF, XHL, JYC, and HHS. YF, RL, AWL, YFF, and XHL wrote the manuscript.

## Conflict of Interest

The authors declare that they have no competing interests.

## Consent for Publication

All authors gave final approval to publish the version.

## Supporting information


**Figure S1.** Subclassification of B cells.
**Figure S2.** Diagnosis and screening of enrolled patients.
**Figure S3.** Clinical efficacy of rituximab therapy on HAM patients.
**Table S1.** Primer/probe sequences.
**Table S2.** Summary of adverse events in all patients who received rituximab.

## Data Availability

Raw scRNA‐seq data analyzed in this study are available in ENA database with the accession ID PRJEB47382. Individual participant data collected during the trials are available for the present study. The study protocol, statistical analysis plan, and informed consent form are also available. Proposals should be directed to corresponding author at fuying@fjmu.edu.cn; to gain access, data requestors will need to sign a data access agreement.
