# Supplementary material for: B‐cell depletion limits HTLV‐1‐infected T‐cell expansion and ameliorate HTLV‐1‐associated myelopathy
Source: Ann Clin Transl Neurol. 2024 Aug 26;11(10):2756–68. doi: 10.1002/acn3.52190 (PMC11514899; doi:10.1002/acn3.52190)
Supplement: Supplementary file 1 — Figure S1. Subclassification of B cells. Figure S2. Diagnosis and screening of enrolled patients. Figure S3. Clinical efficacy of rituximab therapy on HAM patients. Table S1. Primer/probe sequences. Table S2. Summary of adverse events in all patients who received rituximab. [file ACN3-11-2756-s001.docx]

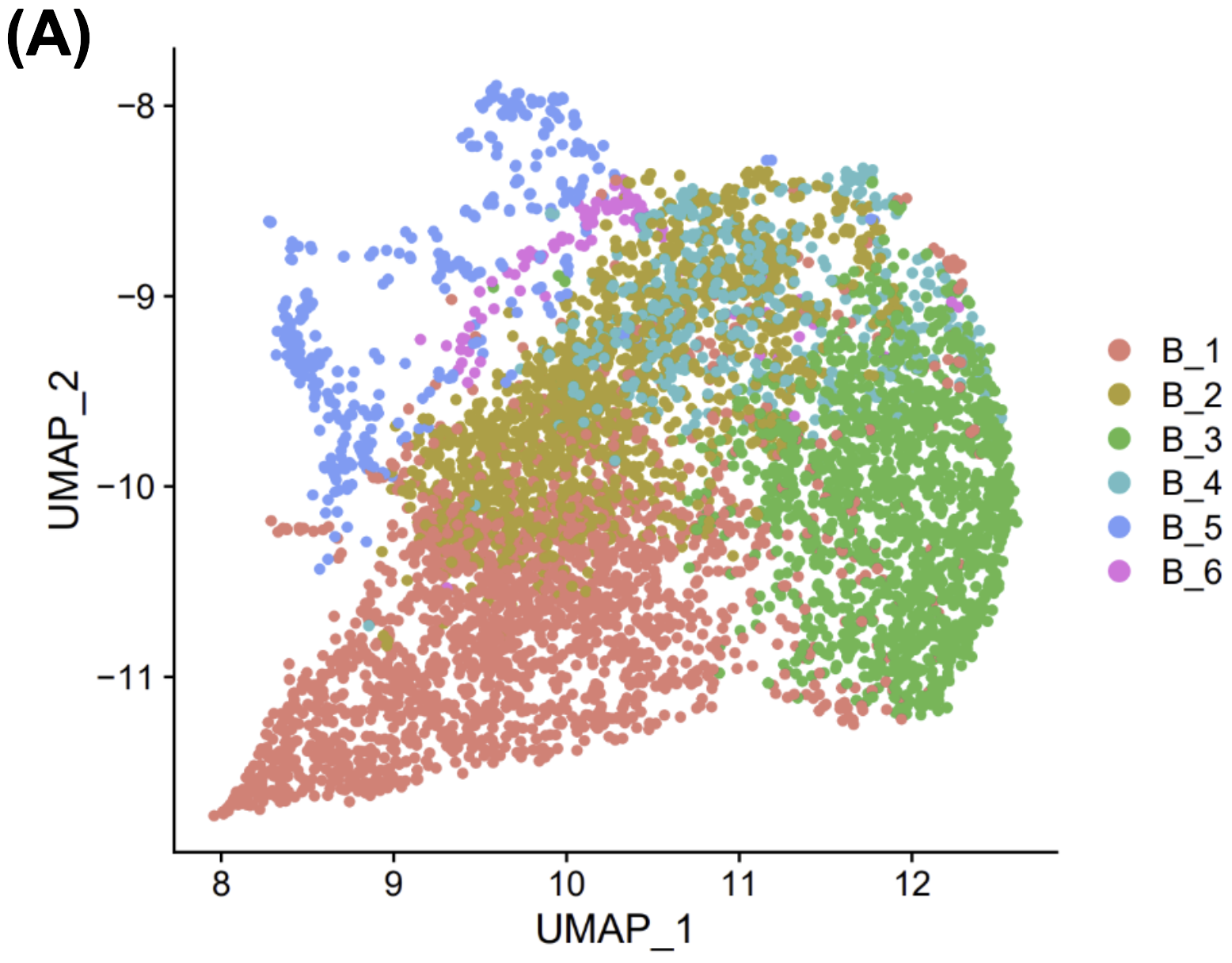


**Figure S1. Subclassification of B cells.**

(A). UMAP visualization of subclassified B cells from all donors, colored by the identified cell cluster.

**
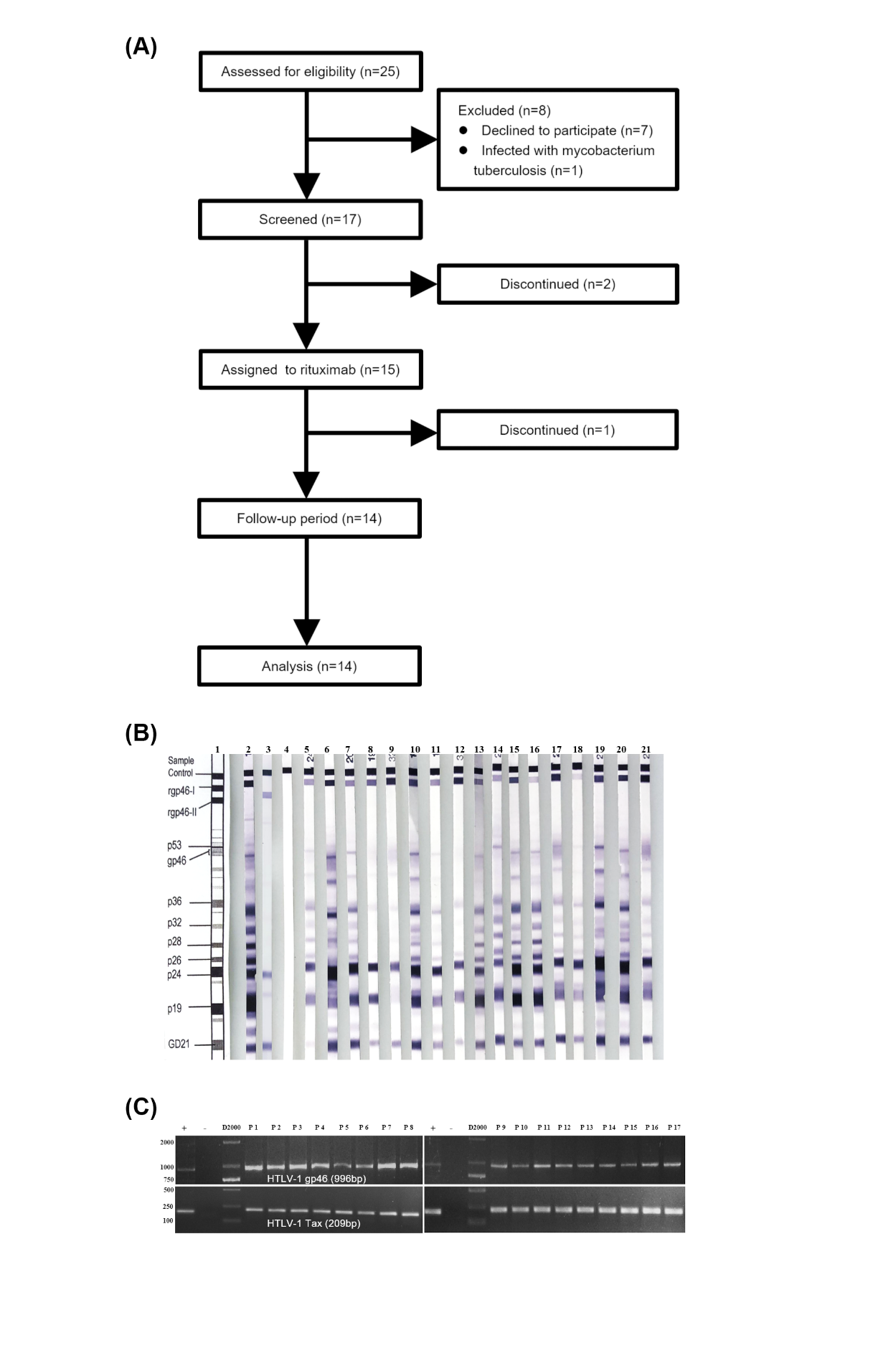
**

**Figure S2.** **Diagnosis and screening of enrolled patients.**

**(A).** Flow chart of screening patients. Reasons for screening failure and discontinuation from the study are indicated. **(B).** Western blot profiles:(1) Identification of HTLV viral proteins bands of MP Diagnostics HTLV Blot 2.4; (2) Strong reactive control I (Reactive for HTLV-I only); (3) Strong reactive control II (Reactive for HTLV-II only); (4) Non-reactive control; (5-21): All strips showed specific band of the HTLV-I recombinant protein rgp46-I, GD21, p19, and p24. CSF samples were obtained from seventeen patients. **(C).** Nested PCR result targeting amplification of the HTLV-1 gp46 gene and Tax gene. Agarose gel electrophoresis demonstrated HTLV-1 gp46-specific band (996 bp) and HTLV-1 tax-specific band (209 bp) obtained from seventeen patients. Recombinant plasmid was used as positive control.


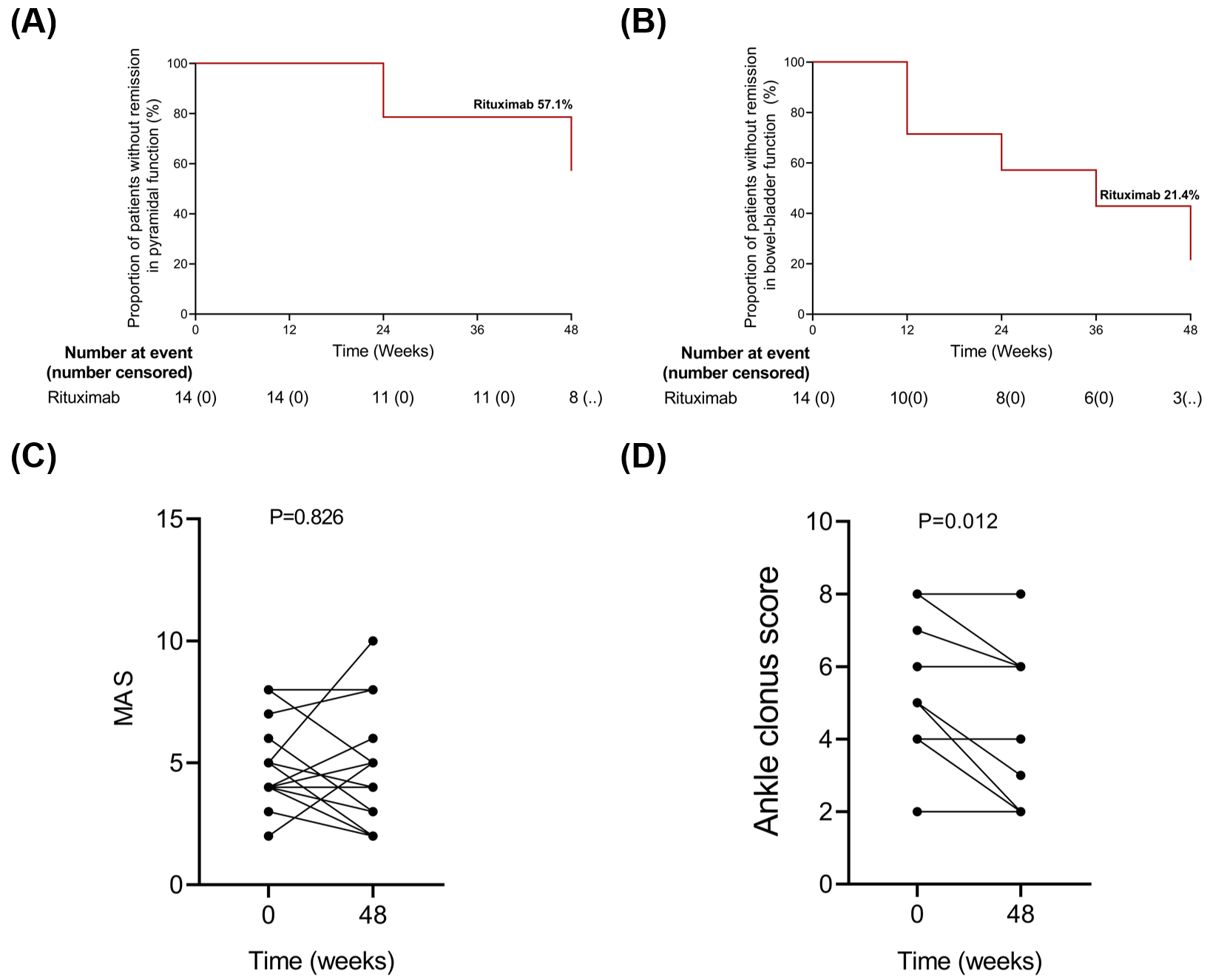


**Figure S3. Clinical efficacy of rituximab therapy on HAM patients.**

The proportion of patients without a decrease at 1-point in pyramidal function score (A) or bowel-bladder function score (B) after treatment with rituximab by survival analysis. Change in MAS (C) and ankle clonus score (D) at baseline and 48-week. Note some patients overlap in the graph.

**Table S1.** **Primer/probe sequences**

| **Gene name** | **Primer sequences** | **Probe sequence** |
| --- | --- | --- |
| HTLV-1 *pX* | Fwd: CCCACTTCCCAGGGTTTGGA | 5’ FAM 3’ BHQ1 |
|  | Rvs: GGCCAGTAGGGCGTGA | CCAGTCTACGTGTTTGGAGACTGTGTACA |
| β-globin | Fwd: TGAAGGCTCATGGCAAGAAA | 5’ FAM 3’ BHQ1 |
|  | Rvs: GCTCACTCAGTGTGGCAAAGG | TCCAGGTGAGCCAGGCCATCACTA |

**Table S2. Summary of adverse events in all patients who received rituximab.**

|  | **Rituximab (n=14)** |
| --- | --- |
|  |  |
| **Total adverse events** | 3 |
| Grade 1 | 3 |
| Grade 2 | 0 |
| Grade 3 | 0 |
| Total serious adverse event | 0 |
| **Most frequent adverse events** |  |
| Infusion reaction | 3 |
| Nasopharyngitis | 0 |
| Headache | 0 |
| Upper respiratory infection | 0 |
| Diarrhea | 0 |
